# Gastric electrical stimulation versus standard medical therapies for long-term symptom control and improved quality of life in drug-refractory gastroparesis patients

**DOI:** 10.1097/MS9.0000000000003321

**Published:** 2025-04-25

**Authors:** Tirath Patel, Muhammad Farhan, Gadeer H. F. Al Shabout, Mustafa Abdulrahman Mohammed, Zaid Abuelata, Abdulaziz Sobhi Shalaby, Abdulrhman Alkassar, Abdelmonkide Ben Khadra, Mariyam M. Kuznetsova, Gayatri Misra, Abhishek Goyal, Fnu Rashi

**Affiliations:** a Department of Neurosurgery, Trinity Medical Sciences University School of Medicine, Ratho Mill Kingstown, Saint Vincent, Saint Vincent and the Grenadines; b Department of Internal Medicine, College of Medicine, Ajman University, Ajman, United Arab Emirates; c Faculty of Medicine, Alexandria University, Alexandria, Egypt; d Department of Internal Medicine, American University of Antigua, College of Medicine, Saint John, Antigua and Barbuda; e Department of Neurology, JFK University Medical Center, USA; f Department of Internal Medicine, Henry Ford Hospital, Clinton township USA

## Abstract

**Objectives:**

This systematic review and meta-analysis evaluated the effectiveness of gastric electrical stimulation (GES) in alleviating symptoms of gastroparesis (GP) compared to alternative medical therapies.

**Methods:**

We conducted a comprehensive search of PubMed, Cochrane Library, and Embase from January 2004 to October 2024 using MeSH terms and keywords related to GP and GES. The search included randomized controlled trials (RCTs) and observational studies published in English. Data extraction followed PRISMA and AMSTAR guidelines. The primary outcome was symptom control, measured using the weighted mean difference and a 95% confidence interval (CI). Statistical analysis was performed using RevMan software, and the certainty of evidence was assessed using the GRADE tool.

**Results:**

A total of 1918 articles were screened, with 4 studies included in the final analysis. The mean difference in symptom control was −0.16 (95% CI: −0.57, 0.26). Heterogeneity was assessed using the chi-square Test, and inconsistency was quantified using the I^2^ index.

**Conclusions:**

GES provides some symptomatic relief in GP, particularly for nausea and vomiting, though the improvements were not statistically significant. Future research should focus on non-crossover RCTs to minimize bias and further explore GES efficacy in idiopathic and postsurgical gastroparesis cases.

## Introduction

Gastroparesis (GP) is a chronic and often incapacitating neuromuscular disorder of the upper gastrointestinal tract^[^1^]^. It is marked by delayed gastric emptying in the absence of any mechanical obstruction. The predominant symptoms include nausea, vomiting, early satiety, postprandial fullness, epigastric pain, bloating, and unintentional weight loss^[^2^]^. Gastroparesis can be idiopathic, or attributable to diabetes mellitus, iatrogenic causes, postoperative states, and post-viral conditions^[^3^]^.

**HIGHLIGHTS**

- Gastric electrical stimulation (GES) offers partial relief for drug-refractory gastroparesis.
- Meta-analysis shows improvements in nausea and vomiting but lacks significance.
- Future studies should focus on idiopathic and postsurgical gastroparesis cases.
- Highlights the need for non-crossover randomized controlled trials to address biases in existing studies.
- GES shows potential in improving quality of life in selected patient groups.

Gastroparesis is diagnosed based on characteristic symptoms once mechanical obstruction has been excluded. Gastric emptying rates can be evaluated using methods such as scintigraphy, the 13C-octanoic acid breath test, or dual-tracer scintigraphy. In cases of mechanical obstruction, the emptying of both solids and liquids is typically delayed, whereas in gastroparesis, the emptying of liquids may still proceed normally.

The pathophysiological mechanisms underlying gastroparesis are multifaceted and involve aberrant gastric motility (including accommodation and emptying), autonomic dysfunction, visceral hypersensitivity, low-grade mucosal inflammation, and cellular alterations in enteric neurons, smooth muscle, and the interstitial cells of Cajal^[^4^]^.

Current therapeutic approaches to managing gastroparesis primarily focus on symptomatic relief and dietary modifications. Conservative management of gastroparesis typically involves dietary modifications and medication^[^5^]^. Generally, patients are advised to consume small, frequent meals high in protein but low in fiber and fat. Prokinetic agents such as metoclopramide, erythromycin, or domperidone are often prescribed. While these medications can help alleviate symptoms, their effect on enhancing gastric emptying remains unclear. Gastric electrical stimulation (GES), also referred to as gastric pacemaker therapy, has emerged as a viable alternative treatment option for patients with refractory gastroparesis who do not respond to conventional medical therapy^[^6^]^. Recent studies have demonstrated that GES provides significant symptomatic relief and physiological improvement through various mechanisms: an early and sustained anti-emetic effect, enhancement of gastric motility in patients with delayed emptying, a prolonged antiarrhythmic effect, late autonomic modulation, hormonal regulation, persistent anti-inflammatory effects, and an overall improvement in health-related quality of life^[^7^]^.

GES provides multiple potential advantages, such as synchronizing intrinsic gastric electrical activity, inducing propagating contractions, and mitigating symptoms in patients with gastroparesis, contingent upon the stimulus settings and stimulation sites. Current pathophysiological research is concentrated on elucidating the causes of disordered motility and visceral hypersensitivity, which contribute to gastroparesis symptoms. Research indicates that patients with gastroparesis exhibit varied clinical outcomes following GES therapy depending on the underlying etiology^[^8^]^. There have been conflicting results from trials of GES for the treatment of symptoms associated with gastroparesis. This review aims to evaluate the effectiveness of GES as a therapeutic option for alleviating gastroparesis symptoms across diverse patient groups, as compared to alternative medical therapies.

## Methods

The work has been reported in accordance with the PRISMA^[^9^]^ and AMSTAR^[^10^]^ guidelines. The study was registered on PROSPERO. The Methods were defined before starting the review process.

### Data collection and search strategy

A computerized search was conducted across PubMed, Cochrane Library, and Embase databases, covering the period from January 2004 to October 2024. The search strategy utilized MeSH terms and combinations of the following keywords: (“gastroparesis” OR “delayed gastric emptying”) AND (“gastric electrical stimulation” OR “electrical stimulation” OR “gastric pacemaker” OR “electrogastrography”) AND (“treatment” OR “therapy” OR “pharmacotherapy” OR “prokinetics” OR “dietary management”) AND (“quality of life” OR “QoL” OR “patient satisfaction” OR “symptom control”). Two investigators independently screened the titles and abstracts and subsequently examined the full texts of the original reports included in the study. Additionally, they conducted further searches for potential studies by reviewing the references of earlier meta-analyses comparing gastric stimulation therapy with other medical therapies or Placebo. Complete data were extracted from these studies for quantitative synthesis.

### Selection criteria

The final inclusion of studies was determined by the consensus of both reviewers. To ensure high quality of evidence and minimal risk of bias, only randomized controlled trials (RCTs) and observational studies were considered. Moreover, studies published in English were considered, while non-English language studies were excluded. The studies adhered to the following pre-specified criteria: (1) Studies involving patients diagnosed with gastroparesis (idiopathic, diabetic, or post-surgical) that is refractory to standard medical treatments. (2) Studies examining the effects of GES as the primary intervention. (3) Studies that compare GES with standard medical treatments, including pharmacotherapy, dietary management, and prokinetics. (4) Studies reporting on long-term outcomes, including symptom control (e.g., nausea, vomiting, bloating) and quality of life measures. (5) Studies were either RCTs, cohort studies, or comparative studies. (6) Studies published in peer-reviewed journals. (7) Studies published in the last 20 years to ensure relevance and up-to-date data. (8) Studies with follow-up periods ranging from 6 months to 5 years to assess sustained effects and symptom control. Additionally, studies with incomplete data were reviewed and discussed; however, they were not included in the formal meta-analysis.

### Study endpoints and definitions

The primary endpoint of this study was symptom control, which includes a reduction in nausea, vomiting, bloating, and early satiety and/or improved quality of life as measured by validated scales (e.g., Gastrointestinal Symptom Rating Scale, SF-36).

### Data extraction

The study’s first author’s name, publication date, design of the study, follow-up duration, and clinical outcomes were systematically reviewed and documented by the reviewers (Tirath Patel and Abdulrhman Alkassar). Any discrepancies between the reviewers were resolved through discussion.

### Quality assessment

Quality assessments were conducted using standardized tools. For RCTs, the Cochrane Collaboration Risk of Bias tool was utilized, which uses 5 different domains (randomization process, deviations from intended interventions, missing outcome data, measurement of the outcome, and selection of the reported result) to calculate the overall risk of bias in the article. All the selected articles were then analyzed by each author and the data was extracted and tabulated in the form of a spreadsheet using Google Docs. All the data entries were then cross-reviewed for any errors.

### Statistical analysis

To measure the primary outcome, we used the weighted mean difference in Symptom control between the Control and Placebo groups as the effect measure. The Confidence Interval was set to be at 95%. A Random Effects Meta-analysis was conducted using the “RevMan” software to obtain a forest plot. The GRADE (Grading of Recommendations, Assessment, Development, and Evaluation)^[^11^]^ was used to assess the certainty of evidence. The tool grades the quality of evidence into one of the four grades; high, moderate, low, and very low, depending upon how close the true effect might be to the estimated effect. A close association means a high quality of evidence and vice versa. The quality is judged for risk of bias, inconsistency of results, indirectness, imprecision, and publication bias. The Chi-Square Test was used to assess the heterogeneity of the study and the values were interpreted according to the Cochrane Handbook of Systematic Reviews^[^12^]^. Inconsistency was quantified using the I^2 index. Values greater than 50 were considered majorly inconsistent, however, the significance of said heterogeneity would depend on the number of studies included, as this is a random effects meta-analysis. For a smaller number of studies, it would not be deemed worthy to perform a subgroup analysis to explore any heterogeneity. Finally, a funnel plot was constructed to visually detect publication bias.

## Results

### Studies included

**Figure 1.**
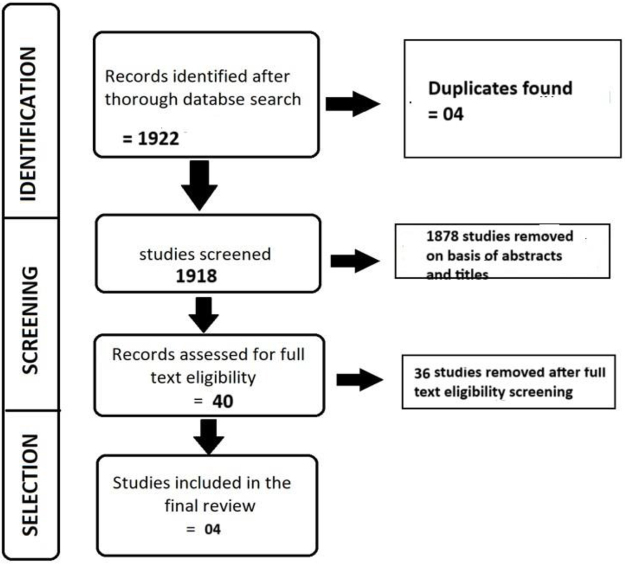
Flow-chart for study selection.

Two reviewers meticulously examined 1918 articles from various databases, focusing on gastroparesis and its symptoms, as well as GES therapy, after removing duplicates. The titles of these publications were independently screened by the two investigators. When the titles met all inclusion criteria and aligned with the research question, the full text was obtained. From the initial pool, 40 articles met the preliminary inclusion and exclusion criteria. Among these, 14 articles were deemed most relevant and were available with abstracts. Ultimately, we included 4 studies^[^13-16^]^ with full texts in the results. Studies without a control group were excluded from the analysis as per the inclusion criteria. The data extraction process is illustrated in the Preferred Reporting Items for Systematic Reviews and Meta-Analyses (PRISMA) flow chart^[^9^]^ (Fig. 1).

### Study characteristics and reported outcomes

We included 4 studies in our systematic review after thorough research. All of these were RCTs with a crossover design. The characteristics of these studies are summarized in the table below (Table 1):

**Table 1:** Characteristics of included studies and reported results

Study, year	Type	number of participants	Reported results (symptom relief)
Ducrotte *et al,* 2019 ^21^	RCT	135	Vomiting score: 2.3 ± 1.7 in experimental group
			Vomiting score: 1.9 ±1.7, *P* = 0.01, in control group (where 0 is daily vomiting, 4 is not vomiting)
McCallum, 2010 ^20^	RCT	36	Crossover phase: vomiting in experimental (ON state) 2.31 ± 1.43
			Vomiting in control (OFF state) 2.03 ±1.48 (where 0 = absent, 4 = severe), median reduction 0%, *P* < 0.001
Abell, 2011 ^19^	RCT	58	An overall treatment effect of a slight, nonsignificant daily decrease in average vomiting scores, −0.12 (−0.26 to 0.03; *P* = .116), was observed by pooling stimulation effects across sessions.
McCallum, 2013 ^18^	RCT	20	Vomiting in experimental group: 2.38 ± 1.24
			Vomiting in control group: 2.71 ± 1.19 median reduction of 17%, *P* = 0.10.

### Risk of bias assessment

Risk of bias tool version 2.0 was used to assess the risk of bias in the included RCTs. Risk of bias (Table 2) was calculated across 5 different domains which were then used to calculate the overall risk for each study by the tool. All 4/4 included trials had a low risk of bias (100%). Each study was checked for bias independently by two authors and no discrepancies were found after mutual discussion.

**Table 2:** Risk of bias

Study ID	Experimental	Comparator	Outcome	Domain 6: overall bias
Abell, 2011	Electrical stimuli	Placebo	Vomiting score	Low
McCallum, 2010	Enterra therapy	Placebo	Vomiting score	Low
McCallum, 2013	Enterra therapy	Placebo	Vomiting score	Low
Ducrotter, 2020	Electrical stimuli	Placebo	Vomiting score	Low

### Effect analysis

**Figure 2.**
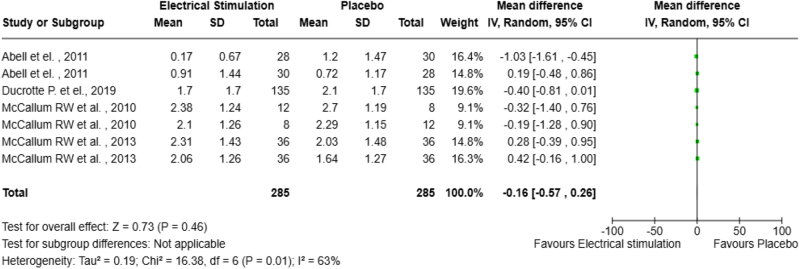
Forest plot.

We constructed a forest plot (Fig. 2) to demonstrate the effect of GES on symptoms of gastroparesis. We found that although patients reported improvement in symptoms such as bloating, vomiting, nausea, etc., the difference was not statistically significant, as shown in the graph below. All the studies included were crossover designs, with a carry-over effect, except for Ducrotte *et al*. Each phase of the crossover has been analyzed individually. Due to the small number of studies included, and the random-effects model used in this analysis, the heterogeneity was not considered worth exploring. The mean difference was found to be −0.16 with a 95% confidence interval of (−0.57, 0.26).

### Certainty assessment

Using the GRADE tool, we concluded that the certainty of evidence to judge the effect of gastric stimulation on symptom resolution is high, i.e., the authors are confident that the true effect is very close to the estimated effect. This was concluded after judging the evidence for risk of bias, imprecision, inconsistency, indirectness, and publication bias.

### Publication bias

**Figure 3.**
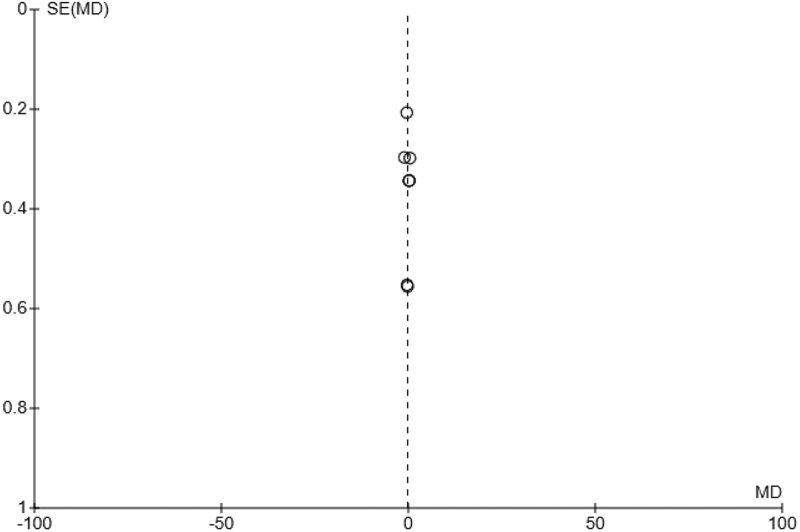
Funnel plot for publication bias.

A funnel plot (Fig. 3) was constructed, which shows high precision, and low risk for publication bias.

## Discussion

Gastroparesis is a clinical condition marked by symptoms indicative of disrupted digestive function in the upper gastrointestinal tract, combined with objective findings of significantly delayed gastric emptying in the absence of mechanical obstruction^[^17^]^. The majority of gastroparesis cases can be categorized into three types: idiopathic gastroparesis (ID, 36%), diabetic gastroparesis (DG, 29%), and postsurgical gastroparesis (PSG, 13%). Idiopathic gastroparesis describes patients who exhibit delayed gastric emptying with no identifiable underlying cause. It is the most prevalent form of gastroparesis, and most individuals affected by ID are typically young to middle-aged women^[^18^]^. A notable subset of ID cases is post-viral, where patients experience a sudden onset of GP symptoms following a viral infection, characterized by severe nausea and vomiting, often improving within a year. Among the various causes of gastroparesis, diabetes is the most commonly recognized^[^19^]^. Other less common causes include Parkinsonism, amyloidosis, paraneoplastic, scleroderma, and mesenteric ischemia^[^19^]^.

Gastroparesis presents with a variety of symptoms such as nausea, vomiting, early feeling of fullness, bloating, belching, and upper abdominal discomfort, which can overlap with symptoms of functional dyspepsia and rapid gastric emptying. Several scales are used to measure symptom severity, including the Gastroparesis Symptom Severity Scale and the Gastroparesis Symptom Index^[^20^]^, which is derived from the comprehensive Patients Assessment of Upper Gastrointestinal Disorders-Symptoms. The revised GCSI-Daily Diary is another tool used in clinical studies to evaluate treatment effects. These scales rely on patient-reported outcomes to gauge symptom severity and the effectiveness of therapeutic interventions.

## Conclusion

In our systematic review and meta-analysis, we discovered that although GES has shown promise in reducing nausea and vomiting, which are among the most troublesome symptoms, along with improvement in overall symptom severity, as reflected in both Gastroparesis Cardinal Symptom Index and Total Symptom Severity scores, the difference was not statistically significant. Not included in the meta-analyses, but some studies showed that patients experienced weight stabilization and gain, particularly those with diabetic gastroparesis. Other studies also showed improvement in bloating and early satiety. However, the results are not statistically significant. Our study has a few limitations. The presence of a carryover effect, due to the lack of an insufficient washover period between the two crossover sessions, leads to the effects of one treatment persisting into subsequent study periods, potentially influencing the outcomes of the next treatment phase. This can lead to biased results, as the observed effects may not solely reflect the treatment being studied but also residual effects from the previous one such as in the study conducted by McCallum *et al*. This compromises the quality of data with the potential for masking the effects of GES. Moreover, the included trials had a small patient population, These smaller samples may fail to accurately reflect the broader population, resulting in heterogeneity within the outcomes, as seen in our analysis. This heterogeneity often stems from individual differences among participants, which tend to have a greater impact in smaller groups. As a result, the conclusions drawn from such studies may lack reliability and fail to be widely applicable.

In the future, we could benefit from conducting more RCTs with parallel arms and not a crossover design to minimize this. Moreover, the presence of a carryover effect and the lack of an insufficient washover period between the two crossover sessions, such as in the study conducted by McCallum *et al*, compromise the quality of data with the potential for masking the effects of GES. In the future, we could benefit from conducting more RCTs with parallel arms and not a crossover design to minimize this. Moreover, most studies involve only Diabetic Gastroparesis patients, so further research is warranted to examine the use of gastric pacemakers in idiopathic and post-surgical cases. Additionally, our review focused on relevant studies published within the last 20 years and excluded pediatric populations and animal studies.

## Data Availability

All the data included in this study are available in this published article and will be provided upon reasonable request.

## Research registration unique identifying number (UIN)

1. Name of the registry: PROSPERO

2. Unique Identifying number or registration ID: CRD42024618319.

## PRISMA checklist

The PRISMA checklist for systematic reviews and meta-analysis was followed. The checklist can be found at: PRISMA (prisma-statement.org) as well.
